# Ionic Compatibilization of Polymers

**DOI:** 10.1021/acspolymersau.2c00026

**Published:** 2022-07-22

**Authors:** Glenn H. Fredrickson, Shuyi Xie, Jerrick Edmund, My Linh Le, Dan Sun, Douglas J. Grzetic, Daniel L. Vigil, Kris T. Delaney, Michael L. Chabinyc, Rachel A. Segalman

**Affiliations:** † Department of Chemical Engineering, University of California, Santa Barbara, California 93106, United States; ‡ Materials Research Laboratory, University of California, Santa Barbara, California 93106, United States; ¶ Department of Materials, University of California, Santa Barbara, California 93106, United States; § Department of Chemistry and Biochemistry, University of California, Santa Barbara, California 93106, United States; ∥ Department of Materials, University of California, Santa Barbara, California 93106, United States

**Keywords:** polymer compatibilization, ionic interactions, ionic cross-linking, copolymer, ionomer, ionic liquid, conjugated polymer, polymer upcycling

## Abstract

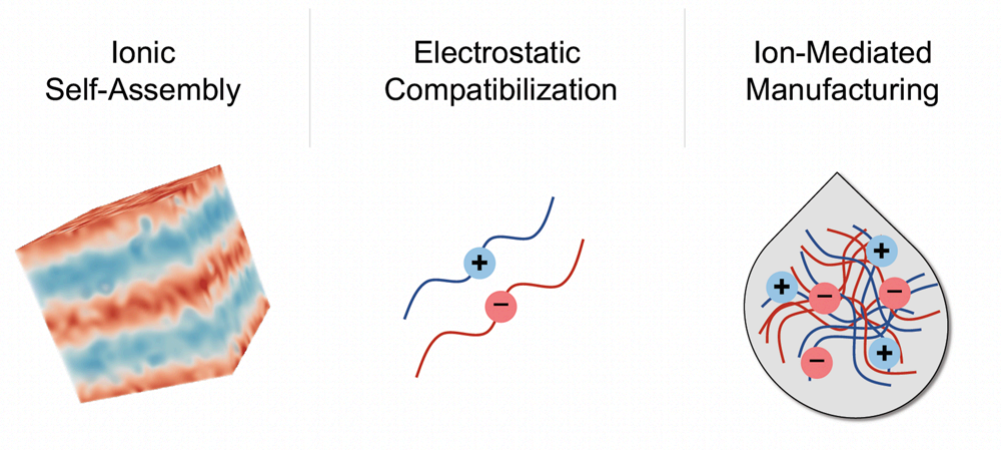

The small specific entropy of mixing of high molecular weight polymers implies that most blends of dissimilar polymers are immiscible with poor physical properties. Historically, a wide range of compatibilization strategies have been pursued, including the addition of copolymers or emulsifiers or installing complementary reactive groups that can promote the *in situ* formation of block or graft copolymers during blending operations. Typically, such reactive blending exploits reversible or irreversible covalent or hydrogen bonds to produce the desired copolymer, but there are other options. Here, we argue that ionic bonds and electrostatic correlations represent an underutilized tool for polymer compatibilization and in tailoring materials for applications ranging from sustainable polymer alloys to organic electronics and solid polymer electrolytes. The theoretical basis for ionic compatibilization is surveyed and placed in the context of existing experimental literature and emerging classes of functional polymer materials. We conclude with a perspective on how electrostatic interactions might be exploited in plastic waste upcycling.

## Introduction

The compatibilization of immiscible polymers has proved to be a challenge since the genesis of polymer science and technology and has re-emerged in recent years as a theme of key importance to the plastic waste problem. Chemically dissimilar polymers are rarely miscible over the full composition range due to an entropy of mixing per unit volume that scales as 1/*N*, where *N* is the degree of polymerization of a chain. Miscible polymer alloys of high molecular weight thus require either remarkable structural similarity (e.g., isotopes or isomers) or specific attractions between dissimilar segments (e.g., via H-bonds) to render the enthalpy of mixing vanishingly small or negative. Truly compatible polymer alloys are rare, and only a few are exploited commercially (e.g., polystyrene/poly(*p*-phenylene oxide)).^1^ More typically, polymer blends exhibit *macrophase separation* and have poor mechanical properties due to narrow interfaces with low chain entanglement between coexisting phases/domains. Other properties, including optical clarity and electronic/ionic conductivity, can be similarly limited by ≳1 μm domains separated by amorphous interfaces. The physical properties of immiscible blends are also unpredictable with melt or solution processing time and history due to a propensity for microstructural evolution.^2^

A variety of strategies have been developed to compatibilize polymer blends, including the addition of a block or graft copolymer to lower the interfacial tension between the coexisting domains, broaden and strengthen interfaces, and stabilize multidomain morphologies against coarsening. In many cases, the block or graft copolymer is created *in situ* by “reactive blending” wherein the copolymer is produced by a chemical reaction between functional groups on the dissimilar polymers. Reactive blending is typically conducted in the melt state within an extruder where mixing and reaction simultaneously occur.^3^

The copolymer that is either blended or reactively formed in an extruder most commonly has dissimilar blocks/grafts joined by permanent covalent bonds or reversible hydrogen bonds.^4^ However, there is a smaller body of literature on copolymers produced by ionic bonds formed by combining acidic units on one polymer (e.g., a sulfonic or carboxylic acid) with basic units on a second polymer (e.g., an amine, pyridine, or imidazole).^5,6^ The proton exchange that occurs between a pair of such units leaves charges of opposite sign on the two polymers, which in a low dielectric environment creates a strong ionic bond. Importantly, no counterions are generated by such a reaction, so the electrostatic interactions are strong and weakly screened. Alternatively, salts of the acid and base functionalized polymers can be used to produce ionic bonding,^4^ leaving residual small molecule counterions in that case. While there is a 30+ year body of literature on ionic bonding in polymers, including well-defined systems with terminal functional groups,^7−14^ we believe this remains an under-exploited and powerful tool for compatibilizing broad combinations of polymers.

**Figure 1:**
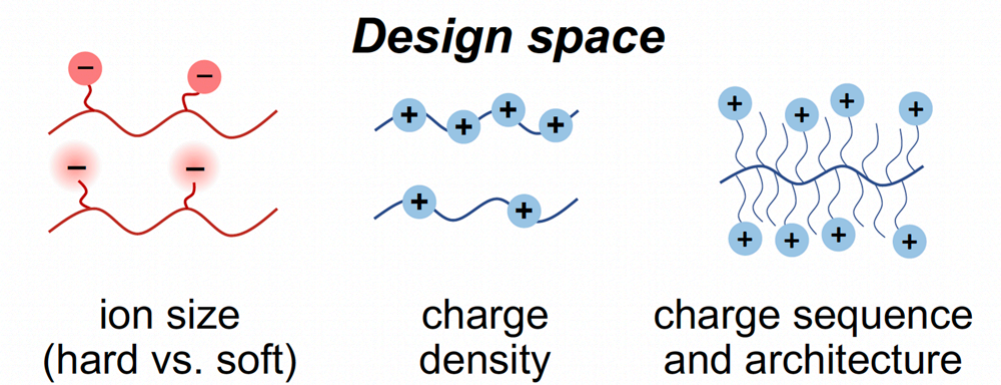
Vast design space of ion-containing polymers spans materials bearing “hard” compact ions or “soft” ionic-liquid-type ions with delocalized charge and variations in both charge density and polymer architecture.

This Perspective addresses the opportunities provided by *ion-mediated, solvent-free* compatibilization of polymers, which is a subject with rich electrostatic physics and materials science that remains largely unexplored from both a fundamental and applied perspective. Polyelectrolytes with high concentrations of ionic groups do not succumb to melt processing, while commercial ionomers can only be processed without solvent because they have very low ion content. There exists a vast materials design space in between with neither low nor high concentrations of ionic groups, which can be further enriched by considering both compact ions, which reinforce and toughen polymers, and bulky, ionic-liquid-type ions with delocalized charge, which plasticize and aid processing (Figure 1). The design space becomes almost limitless when variations in polymer architecture are considered. Furthermore, ionic compatibilization provides opportunities to bring together functional polymer components that are normally difficult to combine homogeneously in the solid state, such as conjugated and nonconjugated polymers and polymers bearing groups that enable responsiveness or switching with external stimuli.

Here, we adopt the term “compatibilization” in the broadest context. This includes full compatibilization, where an immiscible polymer pair is rendered fully miscible by ionic modification, resulting in a single homogeneous phase with only short-range order. An intermediate scenario is that ionic functionalization stabilizes an ordered microphase or a disordered microemulsion with a mesoscopic structural scale (typically 1 nm to 0.1 μm) independent of system size. A lower level of compatibilization corresponds to a situation where coexisting macrophases still exist; however, the interfaces are reinforced by ionic bonds, and both interfacial tension and domain size are reduced.

Another goal of this Perspective is to translate contemporary theoretical descriptions of aqueous polyelectrolyte complexation (e.g., complex coacervation)^15^ to solvent-free blends of oppositely charged polymers. An important issue is whether such systems are best described by polyelectrolyte-inspired theory^16−19^ or by modern theories of supramolecular assembly.^20−25^ We begin with these theoretical considerations.

## Theoretical Considerations

Our initial focus is an idealized melt blend of two polymers, “A” and “B”, that is symmetric in the degree of polymerization, *N*_A_ = *N*_B_ ≡ *N*, statistical segment length, *b*_A_ = *b*_B_ ≡ *b*, backbone dielectric constant ϵ_A_ = ϵ_B_ ≡ ϵ, and charge density, σ_A_ = −σ_B_ ≡ −σ. We imagine that the negative charges on the A polymers originated from the removal of protons from a polyacid and the positive charges on the B polymers arose from proton transfer to a polybase, so that there are no small counterions present. The total charge of each A chain is *Q*_A_ = −σ*N* (in units of the elementary charge *e*) and *Q*_B_ = +σ*N* = −*Q*_A_ is the total charge of each B chain. It should be emphasized that ϵ denotes the static dielectric constant of the polymer backbones in the absence of the charged residues. By construction, such a system is electrostatically neutral if the chains are blended in equal numbers *n*_A_ = *n*_B_ ≡ *n*. This implies a stoichiometric balance of acid and base residues, which is ideal for electrostatic compatibilization. We will defer the discussion of nonstoichometric and nonsymmetric blends to the end of this section.

### Polyelectrolyte Description

The idealized polyacid/polybase blend just described is similar to models of complex coacervation in aqueous mixtures of oppositely charged polyelectrolytes.^26−29^ Such models include the solvent (water) implicitly or explicitly and usually fix the dielectric constant to a large value (ϵ ≈ 80) representative of water. In the simplest models, the water-mediated interactions between polymer segments are not distinguished by segment type, resulting in complex coacervates that are compositionally homogeneous. However, recent theoretical work has predicted that sufficiently incompatible polymer backbones can produce coacervates that are *microphase separated* due to a balance between dissimilar backbone repulsions and electrostatic attraction.^16,17^ The same models can be adapted to solvent-free melt blends of oppositely charged polyelectrolytes,^18,19^ which is the situation of interest here.

A remarkable feature of a stoichiometric blend with polymer components bearing opposite charge is that *macrophase separation is impossible*. This is because a melt that is phase separated on a length scale *L* into negatively charged A-rich domains and positively charged B-rich domains has an electrostatic energy per unit volume proportional to *L*^2^. The electrostatic energy would diverge as the domains coarsen to macroscopic scales, so only homogeneous phases or microphases are possible if all chains are charged and no counterions are present. Thus, oppositely charged blends with incompatible backbones can behave as block copolymers due to attractive electrostatic correlations, even without explicit bond formation, whether covalent or ionic in nature. The propensity of such blends to microphase separate was predicted theoretically many years ago,^30^ but the versatile and tunable nature of such systems has been revealed by recent theory^18,19^ and remains under-exploited in our opinion.

A typical approach in polyelectrolyte theories is to smear the charge uniformly along the A and B chains, so that each segment carries a fractional charge of +σ or −σ. In such a *smeared-charge model* with unfavorable contact interactions between dissimilar polymer segments described by a Flory parameter χ and the melt taken to be incompressible, the random phase approximation (RPA) can be used to locate the critical segregation strength (χ*N*)_c_ for microphase separation to occur.^18,19^ This mean-field prediction can be expressed as
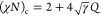
1where *Q* = σ*N* is the magnitude of the total charge per chain and γ is a dimensionless “electrostatic strength” parameter^31^
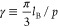
2where *l*_B_ = *e*^2^/(4πϵ_0_ϵ*k*_B_*T*) is the *Bjerrum length* in a medium of relative dielectric constant ϵ (ϵ_0_ is the free space permittivity). Physically, *l*_B_ is the distance of separation between two point charges in a medium of dielectric contant ϵ where the Coulomb energy falls to the thermal energy *k*_B_*T*. The parameter *p* = *v*_0_/*b*^2^ (with *v*_0_ being the segment volume) is the *packing length* introduced by Fetters et al. to correlate polymer entanglement molecular weight and rheology.^32^

Equation 1 sensibly predicts that a blend of chains carrying no charge (*Q* = 0) phase separates at (χ*N*)_c_ = 2, a threshold familiar from the simple Flory–Huggins theory for a symmetric, neutral blend.^33^ Only in this limit does the phase transition correspond to a macrophase separation. For any *Q* > 0, the model can only support homogeneous phases for χ*N* < (χ*N*)_c_ and a lamellar mesophase for χ*N* > (χ*N*)_c_. We can gain an understanding of the magnitude of (χ*N*)_c_ by considering extremes of the electrostatic strength parameter γ. Most polymers have a packing length of order 3 Å, while *l*_B_ ranges from 7 Å in a high dielectric medium like water at room temperature to about 280 Å in a hydrocarbon fluid or polymer (with ϵ ≈ 2) at the same temperature. Over the same extremes in a dielectric environment, γ ranges from about 2.5 in water to about 100 in oil, the latter value most relevant to polymer melt compatibilization. With γ = 100, eq 1 implies that even one charge per chain (*Q* = 1) leads to a moderately high critical segregation strength of (χ*N*)_c_ ≈ 40, while 10 charges per chain would predict (χ*N*)_c_ of ≈400, almost certainly suppressing microphase separation completely. According to such a model, electrostatic interactions are a remarkably powerful force for polymer compatibilization.

The same RPA calculation provides an estimate of the domain spacing *D*_0_ of the lamellar mesophase at the order–disorder transition (ODT), which scales as , where *R*_g_ = *b*(*N*/6)^1/2^ is the unperturbed radius-of-gyration of a chain. The domain spacing is seen to diverge for *Q* → 0, so there is a smooth crossover to macrophase separation in the uncharged case. A very swollen lamellar phase at small *Q* would theoretically unbind due to thermal fluctuations into a bicontinuous microemulsion, but the practical lower limit of *Q* = 1 charge per chain and range of γ values makes this unlikely.

The above predictions based on a smeared-charge model are sensible if γ is  to , i.e., a high dielectric medium. However, they are not reliable in the typical low dielectric case of . Physically, γ can be viewed as the electrostatic energy (in *k*_B_*T* units) required to move two opposite charges from close contact (separation *p* ≈ 3 Å) to infinite separation. Thus, approximately 100 *k*_B_*T* is required to separate two opposite charges in a hydrocarbon polymer melt. In such a case, the weak correlations assumed in the RPA analysis break down and the *opposite charges bind into pairs*, invalidating the smeared-charge assumption. Indeed, in the smeared-charge model, a composition inhomogeneity *must* be accompanied by charge separation, which leads to an overprediction of the microphase separation threshold by eq 1.

**Figure 2:**
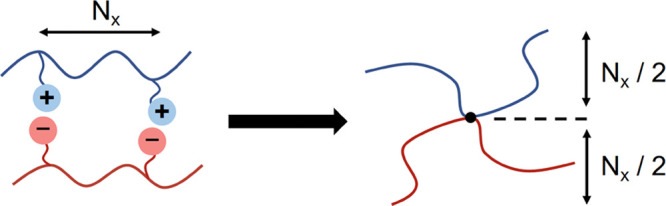
An ionically cross-linked blend with the degree of polymerization between cross-links of *N*_*x*_ has similar microphase separation behavior as an A_2_B_2_ star block polymer with A and B arms of length *N*_*x*_/2.

An improved model of ionic compatibilization for the  case accounts for both the discreteness of charge placement and the propensity for strong ion binding. If the ions are assumed to be regularly spaced along the polymer backbones with *N*_*x*_ = *N*/*Q* being the degree of polymerization between charges and we assume that all ions bind to another one of opposite sign, the melt can be viewed as an ionically cross-linked blend with strand length *N*_*x*_ between the cross-links. Theories of such cross-linked blends^34^ and AB block polymer melts^35^ reveal that the threshold for microphase separation can be anticipated from that for a star copolymer melt with the same structural motif, namely, an A_2_B_2_ star melt with arm length *N*_*x*_/2 as shown in Figure 2. The ODT for such a symmetric star copolymer melt is^36^, which translates to
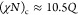
3This expression, incorporating discrete charges and strong ion binding, should be compared with eq 1 on the basis of the smeared charge model. We see that the γ dependence is suppressed in eq 3 and the ODT threshold is a factor of approximately 4 below that predicted by eq 1 for γ = 100. Nonetheless, the critical segregation strength rises linearly with *Q*, so only a few charges per chain are anticipated to affect complete compatibilization for most polymer pairs.

We emphasize that eq 3 is a highly simplified description of the ODT threshold for an ionically bound network, neglecting quenched disorder associated with ion placement on the chains and trapped entanglements. Such disorder likely raises the prefactor above 10.5 and disrupts quasi-long-range lamellar ordering. Nonetheless, when χ*N* exceeds the critical threshold and microphase separation occurs, the physical picture is quite different than in the smeared model case. The individual A- and B-rich domains *do not carry net charge*, but rather, the neutral ion pairs concentrate in the interfaces between domains. At the ODT, the domain spacing is expected to be proportional to the radius-of-gyration of a strand between cross-links, . A more refined theoretical treatment would include dipole–dipole and higher-order multipolar electrostatic interactions among ion pairs, which can lead to ion clusters commonly referred to as “multiplets”.^37^ In the present context, we expect multiplets to be localized in the interfaces between A and B domains and their equilibrium size to reflect a balance of electrostatic attraction against reduced conformational entropy. More theoretical attention is clearly needed on this issue.

### Supramolecular Description

The model just described with discrete charge and ion pairing represents a significant improvement over the smeared charge approach for low dielectric polymers with . Nonetheless, the model is still deficient if charge is installed on the two polymers using acid and base chemistries. While we expect that virtually all ions present in the blend will be paired due to the ∼100*k*_B_*T* energy penalty for separation, it is possible that the forward proton transfer responsible for charging a pair of acid and base units can be reversed. If this occurs, the resulting neutral units are free to separate and the ionic bond is lost. The acid and base groups can then find other complementary partners with which to form new ionic bonds. From this perspective, ionic compatibilization should be treated as a reversible proton transfer reaction between two close acid and base units with an associated equilibrium constant *K*. Such a description falls in the realm of *supramolecular* polymer science.^38^ In the limit that *K* → ∞, all acid and base units are charged and paired and the description of the previous section is recovered. However, at small to intermediate *K* values, many of the functional units remain neutral and a more sophisticated theory is required to deduce phase behavior and degree of compatibilization.

**Figure 3:**
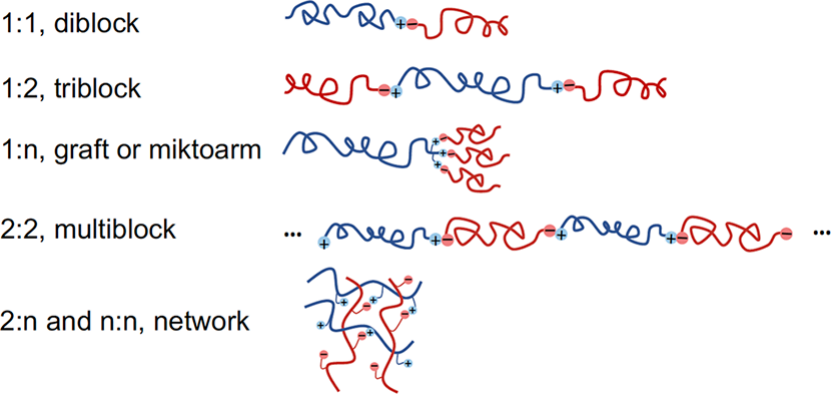
Schematic representation of five types of ionic supramolecular block copolymer structures.

A variety of theoretical approaches have been proposed for treating the simultaneous self-assembly behavior and reaction equilibria of supramolecular polymer blends. A theory by Huh and Jo^20^ used a weak-segregation RPA technique^39^ in tandem with an approximate treatment of reaction equilibria to address the case of monofunctional A polymer blended with a difunctional B polymer, the functional groups located at the chain ends. Such a “1:2” system (see Figure 3) has two products arising from the reversible reaction, A–B diblocks and A–B–A triblocks. Subsequently, Feng et al.^21^ and Lee et al.^22^ developed a self-consistent field theory (SCFT) approach using auxiliary fields for 1:1 and 1:2 systems that avoids the weak-segregation approximation and exactly imposes chemical equilibrium. The 1:1 system is a blend of monofunctional A and B polymers whose only reaction product is an A–B diblock copolymer (Figure 3). This framework was subsequently extended to a 2:2 blend of A and B telechelic polymers^23^ and an *n*:*m* blend^24^ of *n*-arm A stars with reactive ends mixed with *m*-arm B stars with complementary end functionality. In the latter two cases, an infinite number of reaction products are possible and the infinite subset that are linear or tree-like was enumerated by integral equations. A recently developed coherent state (CS) field-theoretic representation^25,40^ allows for a complete enumeration of all reaction products, including rings and networks with embedded cycles. The CS approach also enables relaxation of the mean-field assumption of SCFT and provides superior numerical efficiency.^41^

**Figure 4:**
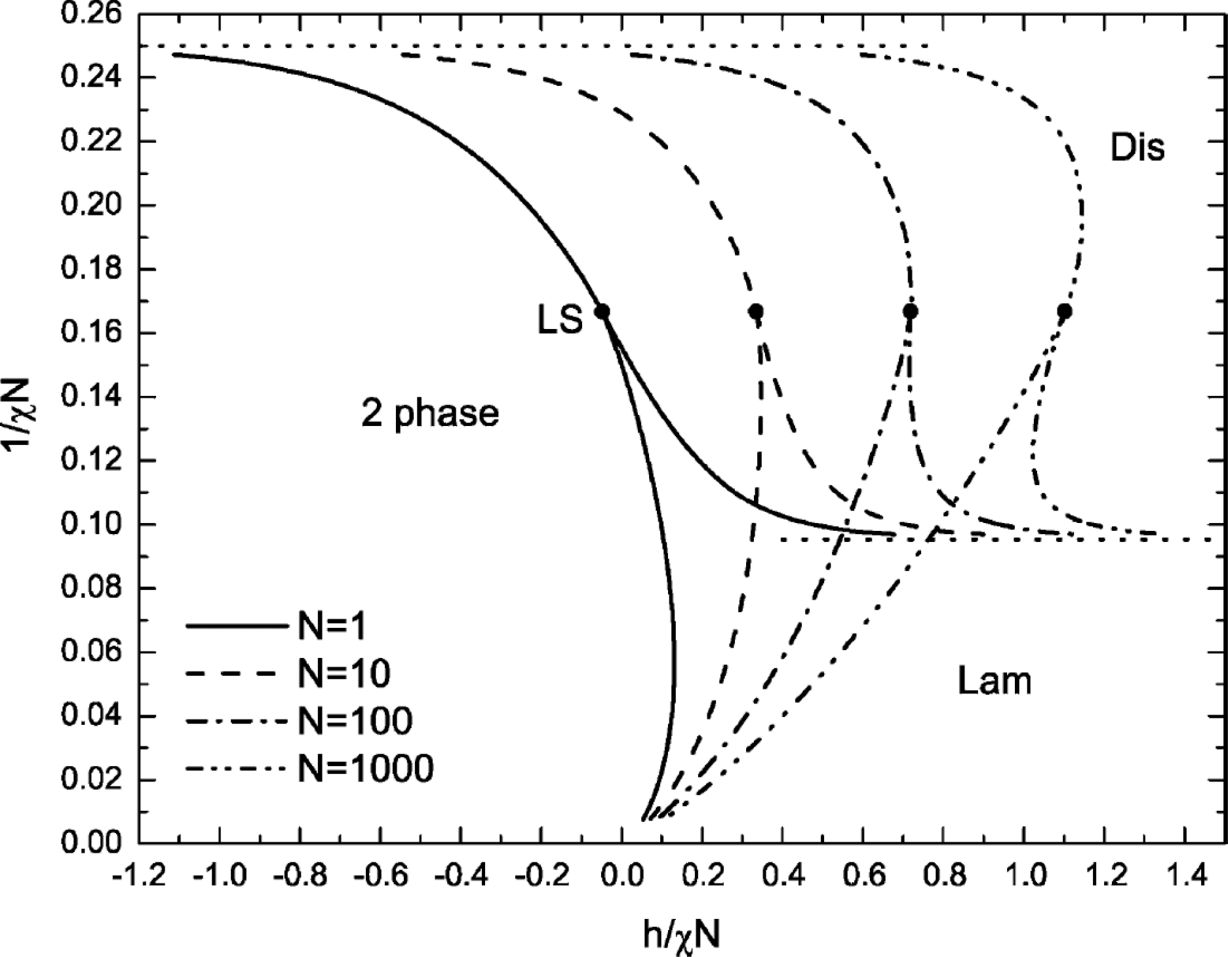
Phase diagram of a symmetric blend of monofunctional A and B polymers (1:1) of length *N*_A_ = *N*_B_ ≡ *N*/2. The inverse segregation strength on the *y*-axis is a pseudo temperature variable, while *h*/χ*N* is an approximately temperature-independent ratio of bond to segregation strengths. The indicated phases are Dis (disordered homogeneous phase), 2 phase (coexistence of two homogeneous liquid phases), and Lam (lamellar mesophase). The points labeled LS are Lifshitz tricritical points. Reproduced from Feng, E. H.; Lee, W. B.; Fredrickson, G. H. *Macromolecules***2007**, *40*, 693–702 (ref (21)). Copyright 2007 American Chemical Society.

An example of a mean-field (SCFT) phase diagram for a symmetric 1:1 blend of monofunctional polymers with equal concentrations of equal length (*N*_A_ = *N*_B_ = *N*/2) A and B chains is shown in Figure 4. The equilibrium constant for the proton transfer reaction between close acid and base groups is assumed to be of the form *K* = (2*v*_0_/*N*) exp(*h*), where *h* ≡ −Δ*F*_b_/*k*_B_*T* is a dimensionless measure of the driving force for ionic bonding, namely, the difference in free energy between two close acid and base reactants and the ion pair product, −Δ*F*_b_, normalized by the thermal energy *k*_B_*T*. The “dilution” prefactor of 2/*N* reflects the fact that only the terminal polymer ends can react.

If *h* and the Flory parameter χ both have predominantly enthalpic contributions, the ratio of bonding to segregation strengths, *h*/(χ*N*), is approximately temperature independent. Figure 4 thus displays the phase behavior of the symmetric 1:1 blend in coordinates of *h*/(χ*N*) vs 1/(χ*N*), the latter a pseudo temperature variable. For weak bonding, only small concentrations of diblock copolymer are produced and macrophase separation is dominant for χ*N* > 4. The case of *h*/(χ*N*) ≫ 1 corresponds to near complete conversion of reactants to diblock product. Here, microphase separation to a lamellar phase occurs at (χ*N*)_c_ = 10.5 in accordance with Leibler’s prediction for a permanently bonded diblock copolymer melt.^39^ The macrophase envelope is seen to expand with chain length, and the Lifshitz tricritical point (LS)^42^ separating critical lines for liquid–liquid macrophase separation and lamellar microphase separation shifts to larger bond strength with increasing *N*. The (mean-field) Lifshitz point does not survive thermal fluctuations but is replaced by a narrow coexistence region of bicontinuous polymer microemulsion.^43−45^ Phase diagrams for symmetric 2:2 and *n*:*n* blends^23−25^ are similar to that for the 1:1 system shown in Figure 4, although additional homogeneous and microphase separated gel phases arise for *n* > 2.^24^ Since the mean-field Lifshitz point delineates competing tendencies for macrophase and microphase separation and hence compatibilization, it is helpful to have a guide for locating it. In symmetric blends with *N* segments on each A or B chain and *Q* acid/base units per chain, the Lifshitz point occurs when *h* = *h*_L_ with *h*_L_ ≈ ln(2*N*/*Q*^2^).

To navigate such phase diagrams, one thus requires estimates of the dimensionless bonding strength *h* or, equivalently, the free energy change Δ*F*_b_ associated with proton transfer. Using conventional acid–base equilibrium arguments,^46^*h* can be related to the difference in p*K*_a_ values of the acid and base residues in the medium, namely,
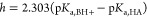
4However, there are two problems with the application of this formula to a proton transfer reaction in low dielectric media. First, experimental measurements of p*K*_a_ are generally not available as a function of dielectric constant.^47^ Even in relatively polar solvents, such as acetonitrile, ion pairs are formed, making the accurate determination of p*K*_a_ very difficult. In low dielectric environments with ϵ ≈ 2, one expects only ion pairs to be present.^48^ Moreover, eq 4 neglects the electrostatic energy of the tight ion pair product. This contribution is known to be important in gas phase proton transfer reactions.^49^ For example, the gas phase reaction of NH_3_ with HCl to produce the ionic solid NH_4_^+^Cl^–^ relies on the favorable electrostatic lattice energy of the reaction product to occur spontaneously.

In spite of the difficulty of estimating *h*, the theoretical phase diagrams suggest that values of *h* of the order of 10–15, i.e., comparable to that of a strong hydrogen bond (10 kcal/mol), are sufficient to compatibilize a 1:1 blend. Such behavior has been reported using strong hydrogen bonding pairs, e.g., 2-ureido-4[1*H*]-pyrimidinone (UPy) and 2,7-diamido-1,8-naphthyridine (Napy), to form polymer blends.^38,50^ Successful melt compatibilization of polymers using low concentrations of strong acid and base groups validate the expectation that ionic bonds in organic polymer media can be at least this strong.^5,7,8^ One also expects a continuum of bonding behavior of acid–base pairs spanning hydrogen bonds to ion pairs depending on the local environment and energetics.^48^ For any given acid–base pair, the resulting behavior can be confirmed using infrared and nuclear magnetic resonance spectroscopy to determine the crossover from hydrogen bonding to ion-pair formation.^51,52^

### Asymmetric Blends

Thus far, we have discussed the idealized case of a binary polymer blend that is symmetric in chain length, charge density, composition, and by implication, charge stoichiometry. Such a system presents the best situation for compatibilization yet is difficult to achieve in practical implementation. In a more typical situation of unequal molecular weights and charge densities, for maximum compatibilizing effect, one should adjust blend composition to achieve charge stoichiometry to avoid “wasting” acid or base functionality. The use of strong acids and bases (*h* ≫ 1) is also helpful to drive the equilibrium conversion strongly toward ionized groups or pairs, and ideally, there should be at least one acid or base residue on each polymer chain to minimize the possibility of macrophase separation. Electrostatically compatibilized blends that are asymmetric have the potential to form a wide range of mesophase structures beyond the lamellar phase discussed here, including exotic Frank–Kasper sphere phases. Moreover, the relative stability and domain spacings of these phases can be tuned continuously using variables such as temperature, dielectric contrast in the two polymer backbones, charge density differences, and added salt.^19^ Self-consistent field theory (SCFT) can be readily applied to such systems using models either of the polyelectrolyte type for systems with high concentrations of bulky, soft ions (e.g., polymeric ionic liquid blends) or supramolecular models for low dielectric systems with small concentrations of compact ions.

### Counterion Effects

Up to this point, we have considered charged blends produced by acid/base proton transfer reactions that yield no small counterions. While this rigorously suppresses macrophase separation if *Q* ≥ 1 and the proton transfer reaction is complete, in many cases, the proton transfer can be reversed at elevated temperature, risking the loss of blend compatibility.

An alternative to acid–base chemistry is to install salt moieties on the two polymers, i.e., −(A^–^C^+^) on polymer A and −(B^+^D^–^) on polymer B, respectively, opening up the possibility of thermally stable ion content in the blend. However, in this case, macrophase separation is possible because the counterions C^+^ and D^–^ can either fail to dissociate or selectively partition in the A-rich and B-rich domains, respectively, to cancel the net electrical charge. Such macrophase separation comes at the price of reduced counterion translational entropy, but it results in a lower enthalpy from reduced A–B segmental contacts.

**Figure 5:**
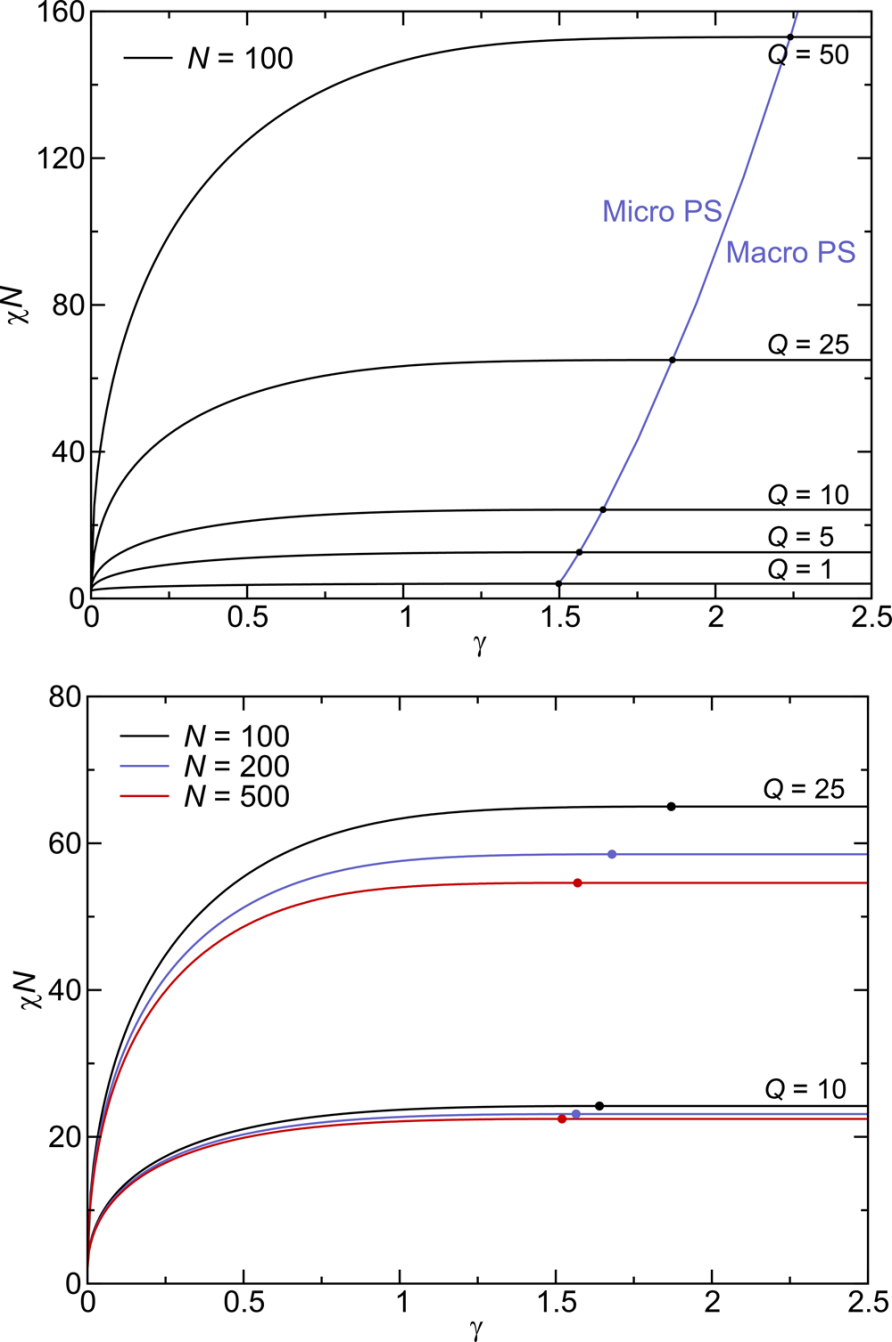
(top) RPA stability analysis of a symmetric blend of chain length *N* = 100 with counterions included for each charged residue. The solid black curves are the stability limits of the homogeneous disordered phase. The segments of the black curves to the left of the points of intersection with the blue curve represent a spinodal instability to a LAM microphase, while the segments to the right describe a critical demixing transition to the coexisting macrophases. The critical segregation strength, (χ*N*)_c_, to either micro- or macrophase separation is seen to grow rapidly with the total charge per chain *Q* and to saturate beyond an electrostatic strength γ of about 1.5. (bottom) Stability curves for three values of *N* and two values of *Q*. The chain length dependence is relatively weak and saturates at large *N*’s.

This entropy–enthalpy interplay and the competition between macrophase and microphase separation can be readily investigated by a RPA, mean-field analysis. For this purpose, we adopt the polyelectrolyte perspective and assume an incompressible symmetric blend with equal chain lengths (*N*’s), equal chain concentrations, and smeared total charges of ∓*Q* on the type A and B chains, respectively. Without any added counterions, eq 1 is easily recovered by the RPA analysis. However, more complex phase behavior emerges if *Q* cationic counterions C^+^ are included with each A chain and *Q* anionic counterions D^–^ are included with each B chain in the alloy. In the top panel of Figure 5, we show the RPA stability limit of the disordered homogeneous phase with counterions included (and *N* = 100) to either macrophase separation (right branch of the black curves) or a LAM microphase (left branch of the black curves). The blue curve traces the locus of mean-field Lifshitz tricritical points^42^ distinguishing microphase from macrophase separation. The critical segregation strength (χ*N*)_c_ is seen to grow rapidly with *Q* and slowly with electrostatic strength γ. For low dielectric polymers with γ > 10, only *macrophase separation* is possible. The lower panel in Figure 5 shows the chain length dependence of the stability limit, which is seen to be weak in these coordinates and to saturate at large *N*’s.

The influence of counterions is thus predicted to be very significant in ionic compatibilization, dramatically narrowing the parameter space for microphase separation. These predictions are consistent with experimental observations of small ion effects.^53−55^ Nonetheless, the electrostatic stabilization of the disordered phase can be large with sufficient bound charge. To the extent that the bound charges and counterions are thermally stable at melt processing temperatures, salt-functionalization can be a useful strategy to affect ionic compatibilization while sidestepping the undesirable reverse proton transfer of acid/base blends.

Finally, we note that our smeared-charge, polyelectrolyte-type analysis neglects ion binding and clustering effects that will occur at large γ. In the case of compact “hard” (or multivalent) ions, the clusters/multiplets can be solid at melt temperature, cross-linking the material and destroying its processability. However, the use of bulky “soft” ions with delocalized charge, such as those that form room temperature ionic liquids, should result in blends with dynamic ionic cross-links that are melt processable. There is evidently an enormous materials design space to be explored as well as the need for a more sophisticated theory that can self-consistently connect ion clustering, self-assembly behavior, and rheology to the local dielectric environment.^56−58^

## Experiments: Compatibilizing the Incompatible

We now turn to experimental realizations of ionic compatibilization, focusing primarily on acid–base chemistries that we have argued have the largest window for suppressing macrophase separation of two dissimilar polymers. Cases of precise polymers with the acid/base functionality at the chain ends are treated first, followed by cases where the functional units are installed as pendants along chain backbones by copolymerization.

### Terminal Functionalization

Russell et al.’s pioneering work^7^ suggested that blending telechelic polyisoprene with tertiary amine functionalities (PI(NR_2_)_2_) and telechelic poly(α-methylstyrene) with carboxylic acid moieties (PαMSt(COOH)_2_) resulted in a multiblock copolymer with ionically bonded junctions between the blocks. The ionic bond formation in this “2:2” system relied on proton exchange during the acid–base reaction, and the resulting supramolecular block copolymer (SBCP) self-assembled into a lamellar (LAM) structure at room temperature. With increasing temperature, the ionic associations forming the copolymer were disrupted, presumably due to reverse proton transfer, resulting in macroscopic phase separation. The corresponding unfunctionalized polymer blend demonstrated upper critical solution temperature (UCST)-type behavior. When the carboxylic acid groups were replaced by stronger sulfonic acid groups (PαMSt(SO_3_H)_2_), a similar LAM morphology was observed, but the ionic association was stable over a wider temperature range, up to 200 °C.

Following this strategy, binary polymer blends with mono-, di-, and multifunctional groups can be used to form SBCPs and mixtures of SBCPs with varying architectures and self-assembly behavior. As shown in Figure 3, there are five combinations that have been explored in the experimental literature to date, 1:1,^10^ 1:2,^11^ 1:*n*,^10,12,13^ 2:2,^7−9,59^ 2:*n*, and *n*:*n*, with *n* > 2.^5^ In the first three combinations (1:1, 1:2, and 1:*n*), the number of supramolecular reaction products are finite, while the latter two combinations (2:*n* and *n*:*n*) can support an infinite set of multiblock chains and network reaction products, including structures with rings and loops (not shown in Figure 3).

**Figure 6:**
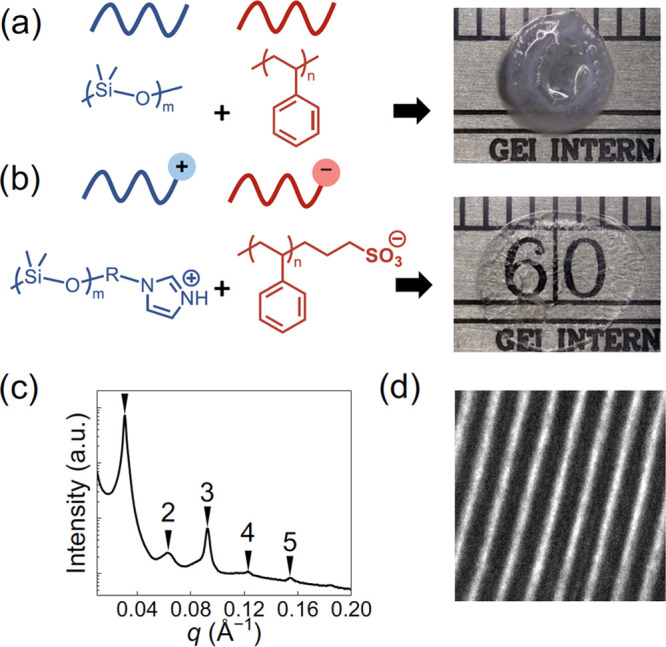
(a, b) Chemical structures of pristine and chain-end functionalized PS/PDMS blends. The reactive PS-acid/PDMS-base blend demonstrates improved optical clarity. (c, d) SAXS and TEM patterns reveal the formation of a LAM microphase.

The most commonly investigated ionic SBCP system involves the polymer pair polystyrene and polyisoprene (PS/PI) with amine and sulfonic acid end groups. Noro et al. prepared a series of PS-SO_3_H (19 kDa)/PI-NH_2_ (17 kDa) blends with various stoichiometric ratios, but a stabilized LAM structure was only observed in the [SO_3_H]/[NH_2_] = 3/1 blend.^10^ This nonstoichiometric bonding behavior was attributed to PI-NH_2_ self-association and aggregation. It is worth noting that the LAM domain spacing *D*_0_ = 48 nm is substantially larger than the corresponding covalently bonded diblock copolymer with similar molar mass (PS20.9k/PI20.5k, *D*_0_ = 28 nm),^60^ suggesting that not all homopolymer chains are bonded, and the free homopolymers swell the corresponding copolymer mesophase. Nevertheless, this diblock-type SBCP (1:1 motif) system with ionic bonding was demonstrated to form a LAM phase as theoretically predicted (Figure 4).^21^ Careful attention must be paid to the selection of end-group chemistry and molecular weight, which determine the bond (*h*) and segregation (χ*N*) strengths. Recently, we observed LAM ordering in a similar 1:1 SBCP alloy of PS-SO_3_H and monoimidazole-terminated poly(dimethylsiloxane), PDMS-Im, as seen both optically and by transmission electron microscopy (TEM) in Figure 6.

**Figure 7:**
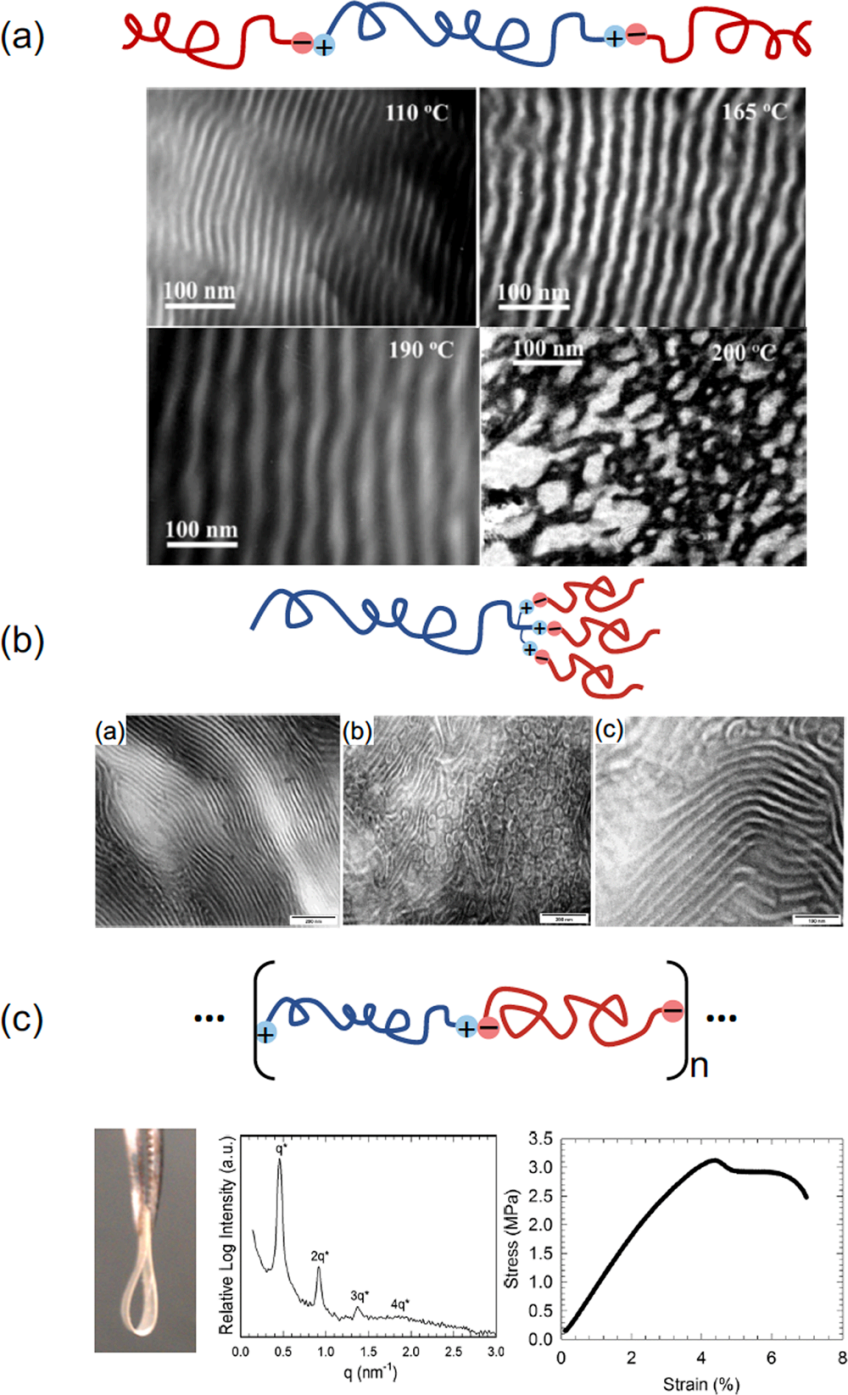
Optical images, TEM, and SAXS analyses of three different SBCPs. (a) Triblock-type SBCPs (1:2 motif) demonstrate an increase in lamellar domain spacing with increasing temperature due to the reversal of proton transfer resulting in nonionic homopolymers that swell the mesophase. Reproduced with permission from Huh, J.; Park, H.; Kim, K.; Kim, K.; Park, C.; Jo, W. *Adv. Mater.***2006**, *18*, 624–629 (ref (11)). Copyright 2006 John Wiley and Sons. (b) Miktoarm star SBCPs (1:3 motif) display defective LAM mesophases where steric constraints might limit proton transfer/ionic bond formation. Reproduced from Pispas, S.; Floudas, G.; Pakula, T.; Lieser, G.; Sakellariou, S.; Hadjichristidis, N. *Macromolecules***2003**, *36*, 759–763 (ref (12)). Copyright 2003 American Chemical Society. (c) Multiblock ionic SBCPs (2:2 motif) produced from PS and poly(isobutylene) telechelics are clear, flexible materials with LAM order. However, the incompletely bonded SBCPs cannot support as much stress as covalently bonded multiblocks, leading to relatively low elongation and stress at break. Reproduced from Zhang, L.; Kucera, L. R.; Ummadisetty, S.; Nykaza, J. R.; Elabd, Y. A.; Storey, R. F.; Cavicchi, K. A.; Weiss, R. A. *Macromolecules***2014**, *47*, 4387–4396 (ref (9)). Copyright 2014 American Chemical Society.

Huh et al. studied the phase behavior of triblock-type 1:2 SBCPs with PS-(SO_3_H)_2_ (14 kDa) and PI-NH_2_ (14 kDa) both theoretically^20^ and experimentally.^11^ A more comprehensive theoretical investigation of the supramolecular triblock blend that did not invoke a weak segregation approximation was provided by Lee et al.^22^ A distinguishing feature of such a 1:2 system is that there are *two* possible supramolecular reaction products: in this case, the diblock PI-*b*-PS and the triblock PI-*b*-PS-*b*-PI. At the stoichiometric condition for triblock formation (67 wt % PI, [NH_2_] = [SO_3_H]), the system self-assembled into a hexagonally packed cylindrical (HEX) morphology, exactly as would be expected from a covalently bonded triblock copolymer of the same composition. When more PS-(SO_3_H)_2_ was added, a HEX to LAM transition was observed in accordance with theoretical predictions.^20,22^ The excess PS-(SO_3_H)_2_ chains presumably form PS-*b*-PI diblocks or remain as a homopolymer, leading to swollen domains and less interfacial curvature (Figure 7a). The LAM domain spacing was observed by Huh et al.^11^ to further increase with temperature, consistent with the expectation that proton transfer is reversible at elevated temperature, leading to a decreased concentration of ionic groups (and bonds) and more free homopolymer of both species. Above 200 °C, the LAM microphase structure was lost and macroscopic phase separation appeared. Such heating-induced macrophase separation can be anticipated by the theoretical phase diagram of Figure 4 for intermediate values of *h*/(χ*N*) ≈ 1 and noting that the 1/(χ*N*) coordinate is an increasing function of *T*.

Blending a monofunctional polymer with a multifunctional polymer (1:*n* motif, each with opposing acid/base functionality) can result in graft/comb or miktoarm SBCPs depending on the number and location of the ionic groups of the later component. Noro et al. blended PS-(SO_3_H)_13_ (23 kDa) with PI-NH_2_ (17 kDa) and observed no evidence of macrophase separation.^10^ Similarly, blending a branched polyethylenimine (1.2 kDa) and a monofunctional PDMS-COOH (1.5 kDa) resulted in compatibilized blends (presumably enriched in graft copolymer) with a LAM microstructure.^13^ A systematic study by Pispas et al. revealed that trifunctional PS-(N(CH_3_)_2_)_3_ and monofunctional PI-SO_3_H blends assembled into LAM or HEX microstructures.^12^ Considering the large conformational asymmetry, the ideal 3-miktoarm star polymer could form a variety of phases with even higher curvature, including Frank–Kasper sphere phases.^61,62^ Nonetheless, in the ionic SBCP system, the average number of bonded PI arms could be lower than three, due to steric hindrance, incomplete proton transfer, or kinetic inaccessibility during processing (Figure 7b).

Blending two telechelic polymers with complementary acid and base end groups (2:2 motif) has gained more attention since the early work by Russell et al.^7^ in the 1980s due to advances in controlled polymerization techniques for telechelic synthesis.^8,9,59^ Conceptually, there are an infinite number of SBCP reaction products in such systems consisting of linear multiblock chains and multiblock rings (cycles) of any length.^23,25^ In spite of this complexity, the equilibrium phase behavior of stoichiometric 2:2 blends with matched chain lengths are qualitatively similar to the SBCP 1:1 phase diagram shown in Figure 4. Nonetheless, the rheological and mechanical properties of 2:2 SBCPs can be markedly different than 1:1 and 1:*n* ionic copolymers. Conventional (covalently bonded) linear A–B multiblock copolymers can demonstrate greater toughness than corresponding diblock and triblock copolymers^63,64^ and have significant potential as compatibilizers^65^ but can be challenging to synthesize with control over both the chain length and concentration of cycles. In comparison, ionic multiblock SBCPs can be prepared relatively easily and demonstrate better mechanical properties than diblock or triblock SBCPs. However, they have a few drawbacks compared to covalently bonded multiblocks. The proton transfer equilibrium results in a large dispersity of the number of blocks per chain, which potentially hinders interconnectivity between domains (chain bridging), influencing both mechanical and interfacial properties. Moreover, in the presence of a large applied force, ionic bonds can be destroyed by either reverse proton transfer or ion pair dissociation, the former being the most likely mechanism. As a result, such ionic 2:2 SBCP materials tend to have a lower elongation and stress at break than comparable multiblock polymers with covalent linkages (Figure 7c).^9^

When the number of terminal ionic groups is enough to cross-link the system (2:*n* or *n*:*n* motif), a supramolecular network is expected. A blend with *Q* ≫ 1 ionic groups per chain is unlikely to achieve the microphase separation threshold of eq 3, instead exhibiting a homogeneous disordered phase. Such a phase would show a diffuse “correlation hole” scattering peak in SAXS analysis but be otherwise featureless. At smaller *Q* and sufficiently large χ (χ ≳ 10.5σ), microphase separation is anticipated. A supramolecular theory for such systems based on blends of A_*n*_ and B_*n*_ stars with complementary acid and base end groups reveals rich phase behavior with gel-point crossovers in network connectivity in both disordered phases and microphases.^24^ Few such systems with well-characterized terminal functionality have been investigated, but we anticipate complex mechanical behavior that can range from melt-intractable materials to blends that can be thermally processed and with solid-state properties that are tunable with acid and base chemistries and the choice of polymer backbones.

### Pendant Functionalization

Copolymerization with monomers that present acid/base or ionic functionality and postpolymerization modifications such as sulfonation provide versatile routes to compatibilizing vast families of polymers. The seminal work by Eisenberg et al.^5^ utilized proton transfer between SO_3_H functional groups created by partial sulfonation of PS or polyisoprene (PI) and 4-vinylpyridine (4VP) groups introduced by statistical copolymerization. Two model systems were employed, namely, blends of lightly sulfonated PS, S-PS, with poly(ethyl acrylate) (PEA) copolymerized with 4-vinylpyridine (4VP), P(EA-*co*-4VP), and blends of sulfonated polyisoprene (S-PI) with P(S-*co*-4VP). Stoichiometric blends of S-PS/P(EA-*co*-4VP) and S-PI/P(S-*co*-4VP) containing 5 mol % or more ionic groups yielded transparent samples with single glass transition temperatures and extended rubbery plateaus.^66^ The single *T*_g_ suggests complete miscibility (i.e., a homogeneous disordered phase rather than a mesophase), whereas corresponding blends without acid and base functionality were opaque and possessed two glass transitions, clear evidence of macrophase separation.

A follow-up study of the P(EA-*co*-4VP)/S-PS system by Douglas et al.^67^ explored a broader range of acid/base functionality, where 5 mol % functional units led to a homogeneous single phase mixture, but at 2 mol %, the blend phase separated into macroscale domains. On the basis of the relatively high polymer molecular weights employed (in excess of 100 kDa), a 2 mol % loading should place multiple acid or base units on nearly every chain (*Q* ≈ 15). If fully ionized, such a system could not macrophase separate. It thus seems likely that the solution casting technique used to prepare the blends did not result in complete proton transfer. Numerous examples can be found in the literature of compatibilizing other types of immiscible polymers with pendant acid and base groups.^53,68−72^ We recently demonstrated compatibilization of the highly immiscible pair polydimethylsiloxane/poly(butyl acrylate) (PDMS/PBA) by pendant functionalization with ionic liquid salt moieties at 10 mol %.

**Figure 8:**
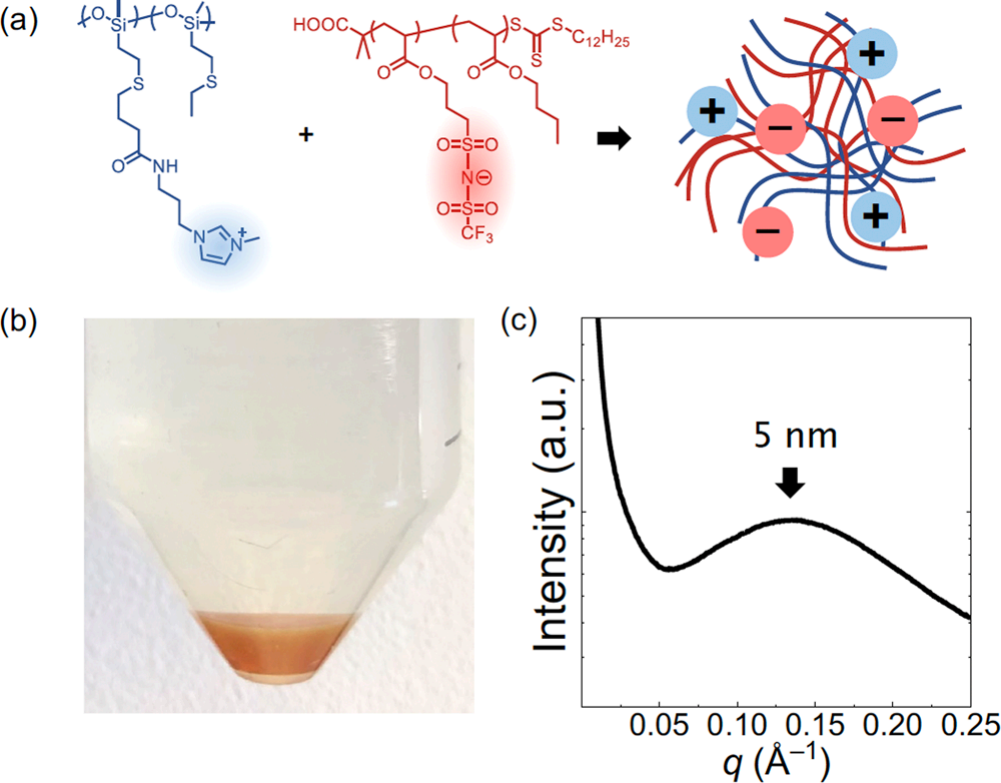
(a) Chemical structures of the PDMS-Im/PBA-TFSI blend, which formed a coacervate (b) in a common solvent. (c) The blend after solvent removal was optically transparent and globally disordered (DIS) but locally segregated with a correlation length of ca. 5 nm.

After mixing imidazolium salt-tethered PDMS (PDMS-Im) and bistriflimide salt-tethered PBA (PBA-TFSI) in solution, a polyelectrolyte coacervate phase developed. The associated counterions were removed by repeated solvent washing and dialysis, and the blend after solvent removal was transparent and exhibited a correlation hole length of ca. 5 nm as demonstrated in Figure 8.

### Conjugated Polymer Blends

The melt intractability and limited solubility of conjugated polymers have restricted the processing of these materials, hindering the fabrication of bulk/shaped structures that are required in various applications such as actuators, bioelectronic scaffolds, or thermoelectric modules. Recently, solvated mixtures of a conjugated polyelectrolyte (CPE) and an oppositely charged insulating polymer have been demonstrated to form fluid- or gel-like complex coacervates that can be processed at very high polymer loading.^73−75^ Electrostatic interactions have previously been utilized to improve the processability of conjugated polymers in water, such as the widely investigated interpolymer complexes poly(3,4-ethylenedioxythiophene)/poly(styrenesulfonate) (PEDOT:PSS) and polyaniline/poly(2-acryl amido-2-methyl-1-propanesulfonic acid) (PANI:PAAMPSA). In these systems, the conjugated monomers are polymerized on the polymer acid templates, where the hydrophilic nature of the template forms a shell that stabilizes the hydrophobic conjugated core in water. Such a stabilization pathway results in the formation of a polymer particle dispersion in water, and the structures of such primary particles are hierarchical, ill-defined, and sensitive to processing conditions. On the other hand, polyelectrolyte coacervation results in the intimate mixing of the two polymers in a fluid or gel state that transforms to an ionically cross-linked solid upon solvent removal.

A conjugated polymer coacervate was demonstrated by Danielsen et al. for a blend of a polythiophene-based CPE with PSS.^74^ Unlike traditional aqueous coacervate systems, the coacervate region emerged upon the addition of organic solvent. This observation suggests the critical role of solvent quality for the hydrophobic π-conjugated backbone in modulating the phase behavior of a conjugated coacervate, likely due to the strong intermolecular interactions between aromatic repeat units that are not present in nonconjugated systems. In a subsequent study by Johnston et al.,^75^ a gel coacervate phase was obtained by mixing a cationic semiflexible donor–acceptor CPE with PSS in aqueous media. Although the phase behavior in this system more closely resembles that of conventional aqueous polyelectrolyte mixtures, the formation of small, liquid-like spherical coacervate droplets was not observed. Instead, the polymer-rich coacervate phase had a colloidal gel structure with the gel modulus enhanced with added salt and the particle size diminished.

**Figure 9:**
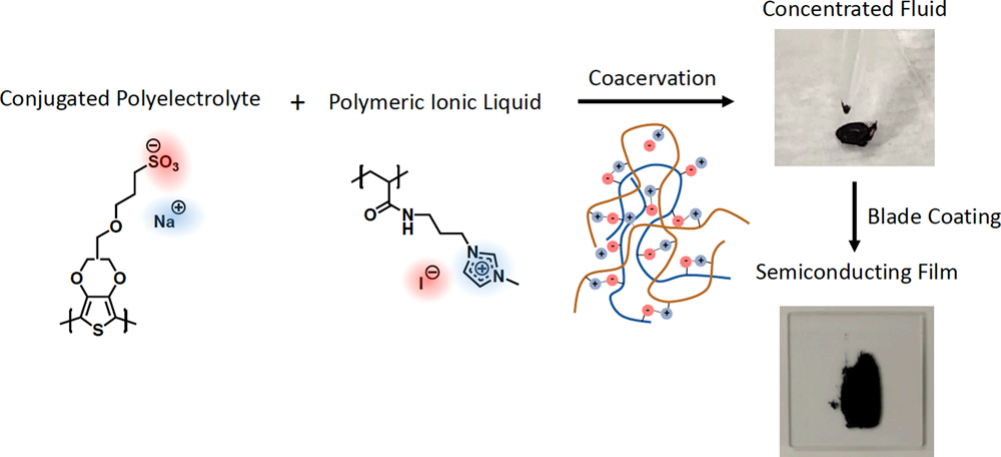
Coacervation between CPE and PIL results in the formation of a concentrated phase with 50% w/v polymer. This paste-like phase can be blade-coated to form a μm-thick homogeneous film with improved electrical conductivity relative to a cast film of the pure CPE component. Adapted from Le, M. L.; Rawlings, D.; Danielsen, S. P.; Kennard, R. M.; Chabinyc, M. L.; Segalman, R. A. *ACS Macro Lett.***2021**, *10*, 1008–1014 (ref (73)). Copyright 2021 American Chemical Society.

Le et al.^73^ provided a demonstration of processing a conjugated coacervate into thick films. In this study, a coacervate between an ethylenedioxythiophene-based CPE and an acrylamide-based polymeric ionic liquid (PIL) with nearly 50 wt % solid loading was blade-coated and dried to form a homogeneous solid film (Figure 9). Due to the high polymer loading, the thickness of the dry film was on the order of 5 μm, which is an order of magnitude larger than film thicknesses obtained from conventional solution casting methods. These studies have demonstrated that ionic functionalization is a powerful tool for processing conjugated polymers at high concentrations, enabling bulk processing of previously intractable materials. Moreover, the strategy appears to be applicable across many different conjugated backbones.

Studies on charged complexes in which both polymers are conjugated, although still scarce, have shown some intriguing complexation physics. CPE–CPE complexes in solid-state precipitate form^76^ and in dilute aqueous solution^77^ were reported to show a qualitative difference in complexation thermodynamics compared to conventional (nonconjugated) polyelectrolytes. At least in the particular systems studied, CPE–CPE complexation appears to have an enthalpic contribution that changes sign with temperature. In particular, at room temperature, complexation is driven mainly by counterion entropy and electrostatic correlations, yet at elevated temperatures, a significant negative enthalpic contribution was measured. This exothermic process was attributed to the extension of the more flexible CPE chains at elevated temperature within the dilute aqueous solution CPE complex, resulting in more delocalization of the π-electron wave function and thus a reduction in chain energy. It is important to note that fluid or gel coacervate phases were not observed in these systems at higher concentration but, rather, solid precipitates were. Such precipitates cannot be easily processed into uniform thick films, although the introduction of an appropriate cosolvent could potentially mitigate this problem.^74^ Together, these observations suggest that charged polymer blend systems with conjugated components have rich complexation behavior that can differ from conventional complex coacervation. The presence of the conjugated backbones in the complex introduces additional intermolecular interactions to the system such as π–π stacking, hydrophobic interactions, hydrogen bonding, and cation−π interactions. A fundamental understanding of how the interplay among such complex interactions determines the phase behavior, structure, and rheology of conjugated charged polymer blends in (lean) mixed solvent conditions is still lacking, and further investigation is needed.

The conformation of the polymer backbone and how chains pack are critical in applications that utilize conjugated polymers, as they play direct roles in determining the material’s electronic structure and transport properties. Some initial insights have been gained on the arrangement of CPE chains within a complex, mainly by exploiting the strong coupling between the conformation of a conjugated polymer and its optical properties. In particular, shifts in optical transitions were used to infer that CPE chains become more planar upon complexation with an insulating polyelectrolyte, likely due to the reduction of torsional disorder.^73,75^ In CPE–CPE complexes with rigidity mismatch between the polymer backbones, a chain extension of the more flexible CPE was observed.^76^ These results underscore the potential of ionic complexation not only to improve the processability of conjugated polymers but also to enhance optoelectronic and transport properties by increasing backbone charge delocalization. However, there is a limited understanding of the nature of chain conformations in conjugated complexes and how factors such as charge density, rigidity mismatch, and molecular structure of individual components influence chain packing and conformation. Moreover, experimental investigations of conjugated polymer complexes to date have not employed CPEs with highly rigid backbones. The flexible chain, mean-field theories outlined here have limited applicability to systems with conjugated components that are locally rod-like and subject to specific and directional interactions such as π–π stacking. Clearly, more theoretical and experimental attention is required, but the universality of complexation-induced conformation changes in such systems may be limited. Other experimental techniques are needed to complement spectroscopic measurements to reveal the chain conformation and structure of the individual components. For example, neutron scattering could be conducted on complexes with one of the chains deuterated to probe the structure of an individual component.

Ultimately, the knowledge of how complexation controls chain conformation and interchain packing of one or more conjugated components will enable electrostatic manipulation of optoelectronic and transport properties for a range of applications. For example, the development of flexible electronics has been challenging due to the high *T*_g_ of the conjugated polymers. Complexing a CPE with an ionically modified elastomer could significantly decrease the material’s modulus, making it more mechanically compatible to soft tissues and enabling the fabrication of stretchable electronic devices for uses in actuators, biointerfaces, or wearable displays. Besides the incorporation of new functionalities, there are also opportunities in optimizing and engineering the existing properties of the conjugated polymers. In particular, electrostatic blends of two CPEs with different backbones could be utilized to continuously tune (with composition and charge density) bandgap and electronic structure for active layers in light-emitting/light-harvesting devices. Currently, electronic structure manipulation of conjugated polymers is limited to the synthesis of new monomers (e.g., donor–acceptor monomers), copolymerization, or postpolymerization functionalization, all of which are laborious, or to doping/additive strategies, which can be unpredictable.^78^

### Plastic Waste Upcycling

Ionic compatibilization can potentially be used to address the growing crisis of plastic waste. An appealing target is mixed polyolefin waste, primarily isotactic polypropylene (iPP) and various grades of polyethylene (PE; e.g., high density PE (HDPE), low density PE (LDPE), linear low density PE (LLDPE), etc.), which represent nearly two-thirds of the plastic manufactured globally and can be relatively easily separated from higher density plastics.^79^ Nonetheless, it is not practically feasible to isolate the individual iPP and PE components in the waste stream. When molten, such mixtures phase separate by polymer microstructure into macroscopic iPP and PE-rich domains.^80^ In the solid state, the materials are brittle and of low value because the interfaces between dissimilar domains are narrow with poor entanglement and few (if any) chains tied into iPP crystals and PE crystals on both sides of the interfaces.

A successful strategy for compatibilizing such blends is by melt compounding with a block copolymer of iPP and HDPE. In principle, a simple covalently bonded diblock copolymer, iPP-*b*-PE, at low concentration (<5 wt %) would provide a significant reduction in interfacial tension, smaller domains, and broader interfaces. Such a compatibilizer is also capable of cocrystallizing with iPP and PE on either side of an interface, yielding tie chains that strengthen and reinforce interfaces in the solid state. Recent work in the Bates and Coates groups have shown that such diblocks are indeed very effective at strengthening iPP/PE alloys, although other copolymer architectures, including linear iPP/HDPE multiblocks and HDPE backbones with iPP grafts, were found to provide superior mechanical enhancement at lower weight fractions.^81,82^

Such a strategy has considerable promise but comes with two drawbacks. The first is cost. Such polyolefin block and graft copolymers are not commercially available and, even if introduced at scale, would be priced as specialty materials likely in excess of $25/kg. With compounding costs included, it is not obvious that the compatibilized iPP/PE alloys could be delivered at a price that the plastics marketplace would tolerate. A second drawback of blending covalently bonded block copolymer is that much of it ends up buried in the bulk iPP and PE-rich domains in micelles and other aggregates,^1,2^ as opposed to being concentrated on the interfacial manifold separating the bulk phases. Consequently, a larger loading of compatibilizer is required, adding to the cost challenge.

An alternative “reactive blending” strategy that forms copolymer by the interfacial reaction of complementary functional groups on the two types of polymers avoids the second drawback described above.^3^ Copolymer is formed only at the interfaces and thus is localized exactly where it can have a maximum effect on interfacial tension reduction and interfacial reinforcement. Only after the interfaces are saturated with copolymer will it migrate via convection and diffusion to the interior of the domains. Compatibilization by reactive blending is widely practiced for many classes of condensation polymers but is rarely used in polyolefin mixtures because of the difficulty of functionalizing saturated hydrocarbon polymers.

**Figure 10:**
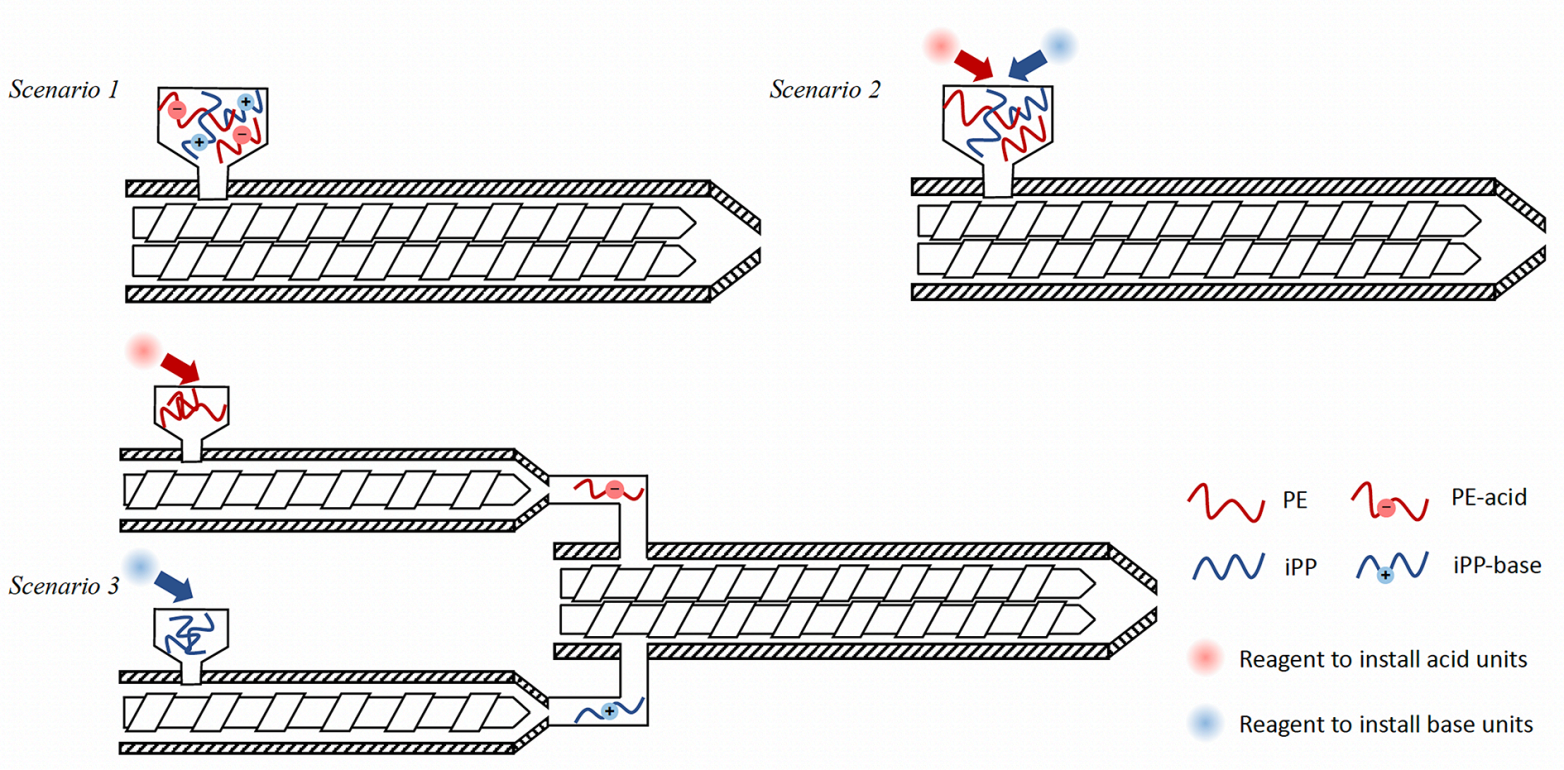
Schematic representation of ionic compatibilization by reactive blending. Scenario 1: a waste stream of prefunctionalized PE-acid and iPP-base is melted and passed through an extruder to affect proton transfer and compatibilization. Scenario 2: PE and iPP chains are selectively functionalized by two regents to install acid and base groups, respectively; the reactions, blending, and compatibilization occurring in a single extruder. Scenario 3: waste streams rich in PE and iPP are functionalized separately in two extruders before being combined in a third extruder.

Recent advances in C–H activation chemistry and other chemistries for polyolefin modification,^83−89^ coupled with an improved understanding of ion-containing polymer thermodynamics, suggest that melt ionic compatibilization of iPP/PE mixtures could be feasible both scientifically and economically. In the most ideal situation, *Scenario 1* (Figure 10), manufacturers of PE would install light acid functionalization of ≪1 wt % on all virgin PE materials and manufacturers of virgin iPP would install comparable levels of base functional units. When a waste mixture is collected, melted, and passed through an extruder, proton transfer can occur between acid and base groups at iPP/PE interfaces, resulting in ionically bonded block or graft copolymer. The choice of acid and base units should be chosen to maximize the stability (irreversibility) of proton transfer at melt processing temperatures. Since macrophase separation need not be fully suppressed but simply interfaces strengthened and domain size reduced, an alternative to acid/base functionalization is to install thermally stable salt units with oppositely signed bound charges on the two polymers (recall the theoretical discussion in Counterion Effects). Waste streams with different concentrations of PE and iPP would be blended together to maintain the stoichiometric balance of bound cationic and anionic groups. Such materials could be repeatedly collected, blended, and recycled into tough, durable products.

The “light” functionalization of ≪1 wt % is mandated to minimize the cost of the reagents needed to functionalize the waste polymers but also to manage the rheology of the compatibilized alloy. Commercial ionomer resins often contain more than 5 wt % of functional groups and remain melt processable. Nonetheless, these typically have alkali or zinc metal counterions, resulting in weaker ionic cross-links in the melt state than in the counterion free case envisioned here. Clearly, the level of functionalization must be maintained either below the gel point of the system or at a level where the dynamic nature of the ionic cross-links is sufficient to enable viscoelastic flow.^58,90^

*Scenario 2* would not require the modification of virgin resins but utilizes two reagents capable of *selectively* attacking PE and iPP chains to install acid and base units, respectively. Both reagents are ideally combined in a single extruder with the molten mixed waste, yielding the compatibilized alloy in a single process step (Figure 10).

*Scenario 3* relies on the existence of separate PE-rich and iPP-rich plastic waste streams. Acid groups would be installed in the PE-rich stream by a nonselective attack in an extruder by a reagent with acid functionality; base groups would be similarly installed on the iPP-rich stream using a separate extruder. The effluent from the two extruders would be combined to form the stoichiometric alloy in a third extruder (Figure 10).

Scenarios 1 and 2 are evidently the most desirable and have the potential for the lowest cost and highest performing PE/iPP alloys. Nonetheless, Scenario 1 would probably require regulatory intervention, and Scenario 2 is scientifically very challenging because most C–H activation chemistries are not selective across PE and iPP substrates. Scenario 3 is the most easily implemented within the current plastics infrastructure. Evidently, the level of ionic modification required for strong interfaces and materials, the chemistries used to install such functionality, and the optimal selection of acid, base, or salt units are subjects that will all require significant fundamental research.

## Discussion and Outlook

Ionic compatibilization of polymers is a subject with a rich history dating back to the 1980s and with great potential for alloying existing and emerging classes of polymers. Nonetheless, it remains a relatively unexploited technique. Many aspects of charge physics that underpin the method remain poorly understood, especially across diverse ion and polymer chemistries, ion placement and concentration, and polymer architecture. It is clear, however, that electrostatic forces can be remarkably powerful in blend compatibilization. As previously discussed, the installation of one opposite charge per chain on two polymers (without counterions) renders macrophase separation impossible, even if the opposite charges do not bind to form ionic bonds. Instead, such a blend can either remain homogeneous or microphase separate into a wide range of nanophase structures. The symmetry and domain periods of the latter can be tuned either with temperature or with salt addition.

An important distinction between electrostatic compatibilization and the more common use of hydrogen bonds to compatibilize polymers^1,4,38,50^ is the breadth of *length scales* over which electrostatic interactions can act. In a high dielectric environment, e.g., an aqueous solution or a melt bearing a high concentration of ionic groups, compatibilization can occur through electrostatic correlations rather than close binding of oppositely charged ion pairs. The correlation (“screening”) length can greatly exceed the ∼10 nm size of a polymer in some instances. At the other extreme of a low dielectric medium, the reversible proton transfer between two close acid and base units (below 1 nm) creates and destroys ionic bonds between polymers, similar to reversible hydrogen bonding between a close donor and acceptor. However, even in this case, the local charging/decharging associated with proton transfer produces long-ranged dipolar modifications to the electrostatic potential, possibly influencing larger-scale structure and thermodynamics.

The use of acid and base functionality to install ionic groups has been emphasized here because it is a simple way to avoid introducing small counterions, which we have argued can promote macrophase separation. Acid/base chemistries also allow for “charging” via proton transfer to occur in either solution or the melt, the latter being crucial for the low cost preparation of alloys in bulk. In spite of these advantages, the thermal reversibility of proton transfer has limited the broad adoption of acid/base ionic compatibilization. More research is needed to find strong acid and base pairs that are stable against reverse proton transfer to melt processing temperatures. The identification of alternative chemistries for installing charges without counterions is another important topic of future research.

In some applications, functionalization by salts consisting of a bound ion/counterion pair can be tolerated. For organic electronic materials, which are generally blended in a solvent environment, the counterions can either be removed by dialysis, left in the blend to modify structure and properties, or exchanged for other ions. Significant freedom exists in such systems to manipulate ion chemistry and size and thereby influence the solution structure and processability of the alloy as well as its solid-state properties such as electronic and ionic conductivity. It is less clear if salt functionalization can be successful in applications such as polyolefin waste upcycling where cost considerations dictate that only a small amount of salt can be tolerated and counterions cannot be removed, yet sufficient ionic cross-links are needed to control domain size and strengthen the polymer–polymer interfaces.

In summary, we believe that ionic compatibilization has great promise and versatility for the creation of new polymer materials. The topic leverages existing knowledge in polyelectrolyte solutions, ionomer melts and solids, and polymer blend phase behavior, but much remains to be understood. A close coupling of theory and simulation with experimentation on well-designed model systems will be necessary to advance the field.
